# Chelation of Ca^2+^ ions by a peptide from the repeat region of the *Plasmodium falciparum* circumsporozoite protein

**DOI:** 10.1186/1475-2875-13-195

**Published:** 2014-05-27

**Authors:** Elena Topchiy, Teresa Lehmann

**Affiliations:** 1 Department of Chemistry, University of Wyoming Laramie, Laramie, WY 82071, USA

**Keywords:** Medicinal chemistry, NMR, Circular dichroism, Malaria, Calcium coordination

## Abstract

**Background:**

Elegant efforts towards the determination of the structural tendencies of peptides derived from the *Plasmodium falciparum* circumsporozoite protein allowed the proposal of a left-handed helical conformation for this protein. The use of circular dichroism and Fourier-transformed infrared spectroscopy applied to various peptides derived from this protein, indicated that they bind Ca^2+^ ions in helical environments. The essential role of calcium in cell function and biological mechanisms is well known. It influences the development of several stages of the *P. falciparum* parasite. However, there is very little knowledge regarding calcium coordination to circumsporozoite proteins. In the present investigation the chelation of Ca^2+^ by the (NANPNVDP)_3_NANP peptide, which contains the first seven 4-amino-acid blocks of the repeat region of the *P. falciparum* circumsporozoite protein, is tested with the use of circular dichroism and nuclear magnetic resonance spectroscopies. Spectroscopy-based solution conformations of the Ca-bound peptide are also determined.

**Methods:**

NMR spectroscopy and circular dichroism were used to test Ca^2+^ coordination by the peptide (NANPNVDP)_3_NANP. Solution conformations for the Ca-bound peptide were determined through molecular dynamics calculations.

**Results:**

The NMR spectra collected for (NANPNVDP)_3_NANP indicate that the signals generated by some of the amino acids located at its C-terminal end are shifted from their original positions upon Ca^2+^ addition. The solution conformations determined for the Ca-bound peptide indicate that the metal ion can be either six- or seven-coordinate.

**Conclusions:**

The investigation described herein strongly supports the coordination of Ca^2+^ ions to some of the amino acids located at the C-terminus of the peptide (NANPNVDP)_3_NANP. The solution conformations determined for the Ca-bound congener of this peptide display many structural features associated to Ca-binding proteins.

## Background

Malaria is one of the most devastating parasitic diseases worldwide. Malaria in humans is caused by one of the four protozoan species of the genus *Plasmodium*: *Plasmodium falciparum, Plasmodium vivax, Plasmodium ovale* and *Plasmodium malariae.* The disease is widespread in tropical and subtropical areas in Africa, Asia and the Americas. The main causative agent of human malaria, the *P. falciparum* parasite, generates approximately 100 million cases and approximately 700,000 deaths worldwide every year [1], mainly affecting children under the age of five. Other risk groups include pregnant women and non-immune adults, such as tourists or military contingent in endemic areas.

Parasite control has had limited success as a result of an increased drug resistance by *P. falciparum.* Immunization is one of the most effective approaches to prevent the disease [2,3]. Some of the current efforts for the development of an effective plasmodial vaccine are focused on the synthesis of peptides that reproduce parts of the circumsporozoite protein (CSP) repeat sequence. The first synthetic peptide vaccine was based on the CSP repeat tetramer Asn-Ala-Asn-Pro (NANP)_3_, the corresponding epitope of the human malaria parasite *P. falciparum*[4,5]. Additionally, phase I clinical trial studies demonstrated that a branched peptide containing minimal T- and B-cell epitopes of *P. falciparum* CSP elicited both antibody and CD4 + -T-cell responses [6,7]. Currently, the most advanced and moderately effective malaria vaccine, RTS,S, is composed of a portion of the central repeat (NANP) and the C-terminal regions of the CSP of *P. falciparum*, linked to the hepatitis B surface antigen [8]. However, this vaccine gives only a 35% reduction in severe malaria in the first year post-immunization.

Although the crystal structures of complete CSPs are not yet available, elegant efforts on the structural direction have led to the understanding of many structural features of these proteins [9-12]. Amongst these efforts, Esposito *et al.* proposed a left-handed helical conformation with βI foldings at all P_i_-N_i+1_ for the CSP of *P. falciparum*[13]. Based on the proposal that helices containing recurrent β-turns can be neutral carriers or pores for Ca^2+^ in biological membranes [14], Verdini *et al*. speculated that the NANP-repeat region of *P. falciparum* CSP could bind calcium ions, and play a crucial role in controlling and regulating many membrane processes during hepatocyte infection by the parasite [15]. Through the use of circular dichroism (CD) and Fourier-transformed infrared spectroscopy applied to CSP peptides with sequences (NANP)_40_, (NANP)_20_, and (NVDPNANP)_3_-(NANP)_3_-NA; Verdini and co-workers concluded that these peptides bind Ca^2+^ ions in helical environments, preferentially at the segment containing the NVDP variant sequences. The essential role of calcium in cell function and biological mechanisms is well known. It influences the development of several stages of the *P. falciparum* parasite [16,17]. However, besides the work of Verdini *et al.*[15], limited progress has been made regarding calcium binding to CSPs. In the present investigation the chelation of Ca^2+^ by the (NANPNVDP)_3_NANP peptide, which contains the first seven 4-amino-acid blocks of the repeat region of the *P. falciparum* CSP, is tested with the use of CD, and nuclear magnetic resonance (NMR) spectroscopies. Molecular dynamics (MD) protocols are used to determine possible solution conformations of the Ca-bound peptide.

## Methods

### Studied peptides

The synthetic peptide (NANPNVDP)_3_NANP, **1,** (American Peptide Company) was examined for Ca^2+^ chelation. Due to the presence of repeating amino acid blocks (units), this peptide was labelled in various positions to facilitate NMR-signal assignment, resulting in the following three alternative versions: Ac-NA*NPNV*DPNANPNVDPNANPNVDPNANP-NH_2_, Ac-NANPNVDPNA*NPNV*DPNANPNVDPNANP-NH_2_, and Ac-NANPNVDPNANPNVDPNA*NPNVDPNANP-NH_2_, where A* and V* denote fully deuterated alanine and valine residues. Once the assignments of all the signals in the NMR spectra were achieved, a non-labelled of version of **1** (Ac-NANPNVDPNANPNVDPNANPNVDPNANP-NH_2_) was used for Ca^2+^ binding studies. In each peptide, the N-terminal amino group was blocked by acetylation and the C-terminal carboxyl group by amidation.

### NMR sample preparation

NMR samples for the peptides under study were 1 mM in 10%D_2_O/90%H_2_O. The pH was adjusted by adding 0.1 M NaOH to a final value of 7.5. A peptide sample in the presence of Ca^2+^ (**1** + Ca^2+^) was prepared by adding 1 molar equivalent of CaCl_2_ to 650 μl of **1** in 10%D_2_O/90%H_2_O (pH 7.5).

### CD sample preparation

The initial apo-peptide sample was 20 μM in water (pH 7.5). A calcium titration experiment was performed by adding 0.5, 1, 2, and 4 molar equivalents of CaCl_2_ (Sigma-Aldrich) to the peptide sample.

### CD spectra collection

Spectra were recorded on an AVIV model 202 circular dichroism spectrophotometer, using a 1-mm path cell at room temperature. Each CD spectrum was an average of 22 scans.

### NMR spectra collection

NMR spectra were performed at 600 MHz in a Bruker Avance III 600 (Bruker BioSpin Corp, Billerica, MA, USA) with a 5.0 mm multi-nuclear broad-band observe probe. All NMR spectra were collected at 278 K and referenced to HDO as the internal standard. Totally correlated spectroscopy (TOCSY) and nuclear Overhauser effect spectroscopy (NOESY) spectra were collected with 100 and 200 milliseconds mixing times, respectively. These spectra were recorded with 512 t_1_ points and 2,048 complex points for each free induction decay. The number of scans per t_1_ point was 32. Water suppression for all samples was performed with the WATERGATE sequence incorporated into all the two-dimensional experiments. Spectra were Fourier transformed using a Lorentzian-to-Gaussian weighting and phase shifted sine-bell window functions. Processing and analysis of the two-dimensional NMR data were performed on an Intel Xeon computer using NMRPipe [18], NMRViewJ [19] and Topspin3.0 (Bruker BioSpin Corp, Billerica, MA, USA) software.

### Structure calculations of 1 + Ca

All calculations were carried out with Discovery Studio 3.1 (Accelrys, San Diego, CA, USA) on an Intel Xeon 5600 series. The intensity of the cross signals detected in the NOESY spectra was classified as strong, medium, or weak on the basis of visual inspection of these spectra. Nuclear Overhauser effect (NOE)-derived distance constraints were set at 1.9-3.0, 2.5-4, and 3.5-5 Å for strong, medium, and weak NOEs, respectively. The distance-dependent dielectric constant algorithm was used with an implicit solvent dielectric constant of 80. Non-bonded van der Waals interaction was cut-off at 14 Å. Molecular dynamics calculations used the Leapfrog Verlet dynamics integrator with a 0.001 picosecond (ps) time step. All energy minimizations and charge assignments used the CHARMm force field. Constant temperature and volume (NVT) with Berendsen thermal coupling was used as the dynamics ensemble for non-periodic systems. Long-range electrostatics was treated with spherical cut-off. The starting extended conformation of **1** + Ca was first minimized by steepest descent method followed by conjugate gradient minimization to a root-mean-square (rms) gradient of < 0.001. The distance constraints were then applied, and the minimization steps were repeated. These structures were used in the molecular dynamics simulated annealing. For each structure five calculations were run starting with a different seed for the initial velocities. The structure was heated and equilibrated over 10 ps from 5 to 1,000 K, with velocities assigned every 0.001 ps. No distance constraints were used in this first step in order to randomize the structure. Molecular dynamics was run for 4 ps, with distance constraints applied with a force constant of 0.06 kcal mol^-1^ Å^-1^. Next, the force constants were scaled to 120 kcal mol^-1^ Å ^-1^ over 7 ps in a series of 0.4 ps molecular dynamics runs. The system was allowed to evolve for 6 ps, and then cooled to 300 K over 7 ps. At this temperature, the force constants were reduced to their final values of 60 kcal mol^-1^ Å ^-1^ over 4 ps in a series of 0.4 ps molecular dynamics runs. The system was allowed to equilibrate for 5 ps, followed by a final 15 ps molecular dynamics run. The coordinates of the final 5 ps of the 15 ps molecular dynamics were averaged and minimized by 1000 steps of steepest descent method followed by conjugate gradient minimization to a rms gradient of <0.01 with distance constraints set to 60 mol^-1^ Å^-1^. The SHAKE algorithm [20] was used to fix all bond lengths to hydrogen atoms.

## Results

### CD spectroscopy

**Figure 1:**
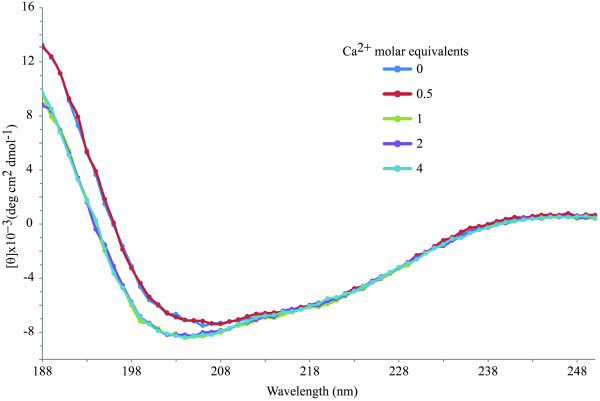
**Circular dichroism spectra.** Titration of **1** with CaCl_2_ performed to determine at which Ca:**1** ratio the reaction between the peptide and the ion is complete.

In order to determine the Ca:**1** ratio required for the NMR experiments, a CD titration experiment of the peptide with CaCl_2_ was performed. As shown in Figure 1, the addition of CaCl_2_ induces a slight change in the CD curve after a Ca:**1** ratio of 1:1 is reached. Further metal additions did not change the CD curve, which indicates the reaction between Ca^2+^ and **1** is complete at 1 molar equivalent of CaCl_2_.

### NMR assignments

Repeating units and amino acids duplication caused the overlap of the NMR signals in the NMR spectra collected for **1**, which required the use of double- and single-labelled versions of this peptide to achieve complete assignments (see Methods). Once the signals for all amino acids in **1** were achieved, the peptide was sequenced through the overlap of TOCSY and NOESY spectra.

**Figure 2:**
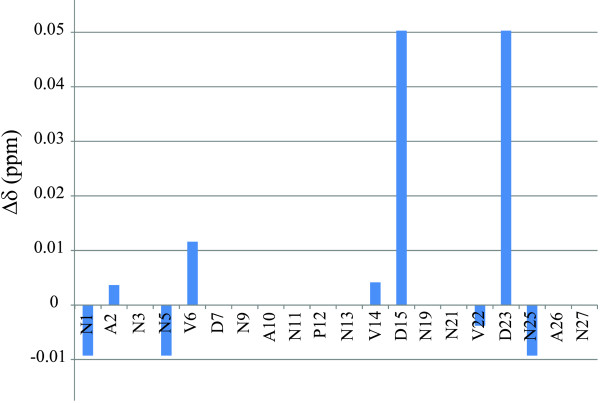
**Differences in chemical shifts between 1 and 1 + Ca.** A positive bar indicates that the chemical shift of **1** is larger. Values are shown for the α-protons.

**Figure 3:**
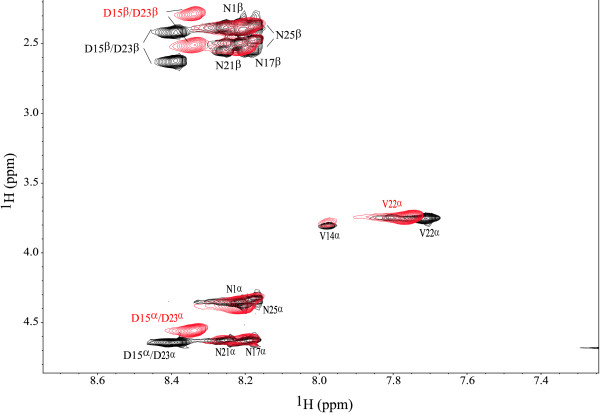
**Overlap of TOCSY spectra.** Portion of the overlap of the TOCSY spectra collected for **1** (black) and **1** + Ca (red) displaying the signals generated by D23, D15, V14 and V22 in both NMR samples.

**Figure 4:**
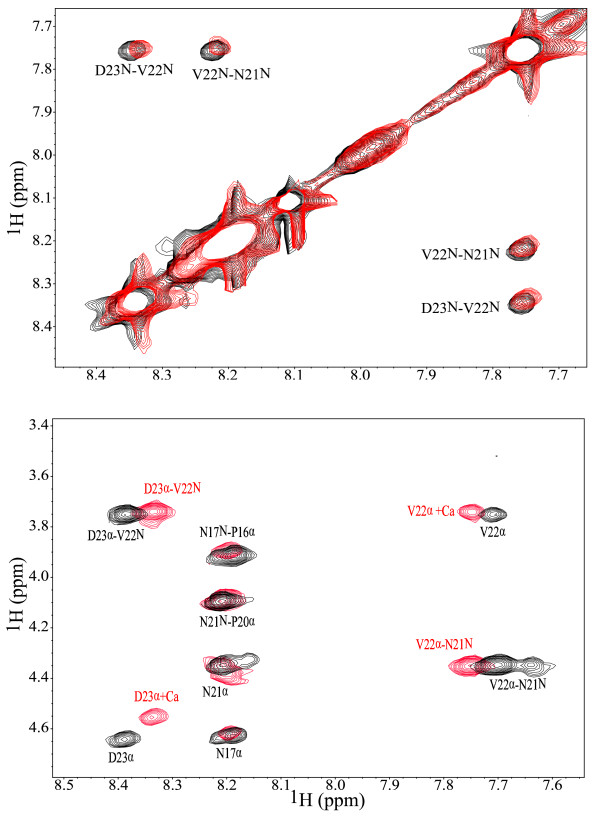
**Overlap of NOESY spectra.** Portions of the overlap of the NOESY spectra collected for **1** (black) and **1** + Ca (red) showing the non-trivial NOEs detected for these peptides.

In order to monitor the effect of Ca^2+^ ions on **1** through NMR, TOCSY and NOESY spectra of **1** + Ca were collected. Addition of calcium to **1** caused a number of NMR signals to shift from their original positions in the apo-peptide spectra. The differences in chemical shifts between the NMR spectra of **1** and **1** + Ca^2+^ are shown in Figure 2. Figure 3 shows a portion of the overlap of the TOCSY spectra of **1** and **1** + Ca^2+^, displaying the signals generated by D23, D15, V14 and V22 in both NMR samples. The overlap of the NOESY spectra collected for **1** and **1** + Ca^2+^ (Figure 4) indicates that no additional non-trivial NOE signals appear as a consequence of Ca^2+^ addition to **1**.

### Molecular modelling

In order to find configurations of **1** + Ca which are compatible with the NMR data generated for this peptide, we calculated solution structures for it assuming Ca binding to happen through residues V14 and/or V22, D15, D23, and N25. Binding through these residues is supported by the shifts of their NMR signals upon Ca addition (*vide supra*). It is also consistent with the fact that many Ca-binding proteins exhibit continuous or semicontinuous binding loops (the majority of the protein ligands to the Ca^2+^ ion are most likely to be part of the same local segment of the polypeptide chain) [21]. For the V residues Ca binding was performed through the peptide carbonyl oxygen atoms. The N and D residues were bound through the oxygen atoms in their side chains. These binding choices were made based on crystal structures reported for many Ca-binding proteins ([21] and references therein). Various coordination alternatives of these residues (Table 1) were tested. The coordination numbers of six and seven were selected based on crystal structures of a large number Ca-binding proteins exhibiting either six or seven ligands to the Ca^2+^ ion. The structure statistics for the coordination alternatives tested is shown in Table 2. These structures are separated in two groups, based on the number of ligands to the Ca^2+^ ion considered.

**Table 1:** Conformations for 1 + Ca

**Conformation/coordination number**	**Ligands**
S1/6	D15, V22, D23, N25, two H_2_0 molecules
S2/6	D15, V14, D23, N25, two H_2_0 molecules
S3/6	D15, V14, D23, N25, V22, one H_2_0 molecule
S4/7	D15 (bidentate coordination), V22, D23, N25, two H_2_0 molecules
S5/7	D15, V22, D23 (bidentate coordination), N25, two H_2_0 molecules
S6/7	D15 (bidentate coordination), V22, D23, N25, V14, one H_2_0 molecule
S7/7	D15, V22, D23 (bidentate coordination), N25, V14, one H_2_0 molecule

Conformations tested in MD calculations for the peptide **1** + Ca including six- and seven-coordinate calcium ions.

**Table 2:** Structure statistics from MD

	**Six-coordinate structures**			**Seven-coordinate structures**			
**Potential energy terms (kcal/mol)**	**S1**	**S2**	**S3**	**S4**	**S5**	**S6**	**S7**
Total	1707.51	1638.72	1248.95	1709.06	1765.77	740.28	521.86
Constraint	1066.73	1821.45	1089.46	1204.44	1163.43	486.12	496.99
Angle	674.57	236.04	508.17	560.38	615.02	505.06	288.13
Dihedral	252.06	160.98	181.92	187.70	199.90	164.5	155.52
van der Waals	-39.53	-134.08	-110.28	-99.54	-123.66	-114.10	-146.59
Bond	260.69	126.79	194.54	282.12	297.20	181.70	184.45
Improper	21.62	5.34	8.64	11.70	7.36	8.97	13.01
Electrostatic	-528.63	-577.81	-623.50	-437.74	-393.48	-492.0	-469.65
Agreement with NOE data (%)	41	38	38	34	47	50	53

Energy terms derived from MD calculations for all the conformations tested for **1** + Ca, and the agreement with NOE data generated through comparisons of the proton-to-proton distances measured for the final structures and the NOE-distance intervals.

The selection of the configurations of **1** + Ca that are most likely to be found in solution is guided by the examination of the following parameters shown in Table 2: agreement with the experimental NOE data, which indicates the configuration that exhibits the lowest number of violations of the NOE constraints; constraint energy, which indicates the configuration for which it is easier to keep its proton–proton distances inside the NOE-distance intervals in terms of energy; and total potential energy, which speaks of the stability of the structure. The number assigned to the agreement with the NOE data is calculated by considering how many of the proton-to-proton distances determined from the calculated conformation remain inside the NOE-distance intervals initially imposed to the structure as NOE-restraints.

**Figure 5:**
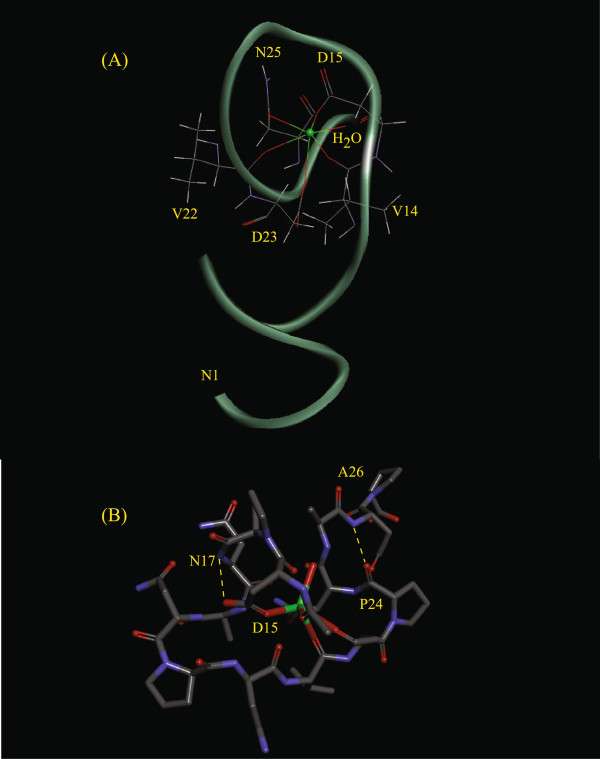
**Peptide conformation S3.** Conformation of **1** + Ca with the lowest potential energy value determined with the Ca ion in a six-coordinate environment. **(A)** Full peptide displaying the Ω-loop involving residues V14-N25. **(B)** Portion of S3 showing the MD-predicted hydrogen bonds originating the Asx turn between residues D15 and N17, and the γ-turn between residues P24 and A26.

**Figure 6:**
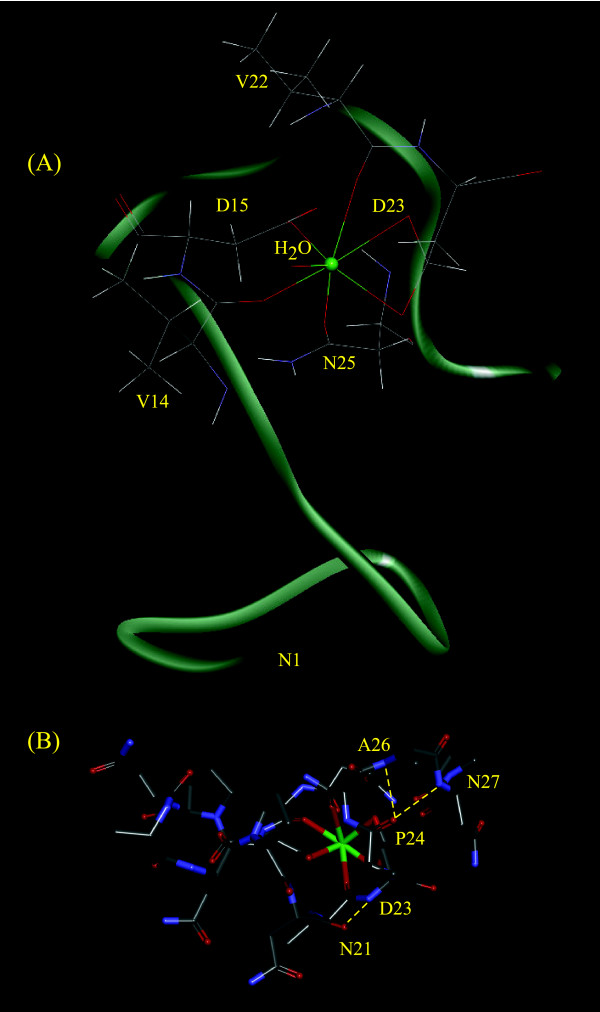
**Peptide conformation S7.** Conformation of **1** + Ca with the lowest potential energy and highest agreement with the NMR data determined with the Ca ion in a seven-coordinate environment. **(A)** Full peptide displaying the Ω-loop involving residues V14-N25. **(B)** Portion of S7 showing the MD-predicted hydrogen bonds originating γ-turns between residues N21 and D23, P24 and A26, and P24 and N27.

Comparison of the results displayed in Table 2 for the six-coordinate configurations indicates that S3 is the most stable (lower total potential energy). Conformations S1 and S3 exhibit similar values for their constraint energies, and the agreement with the NOE data is similar for all conformations in this group. Based on these results, we favor S3 as the most likely configuration for **1** + Ca among the six-coordinate alternatives on the basis of its low total potential energy. Comparison of the molecular dynamics results obtained for the seven-coordinate alternatives indicates that S7, with its low total potential energy and high agreement with the NOE data, should be selected as the most likely configuration for **1** + Ca among the seven-coordinate alternatives. Conformations S3 and S7 are shown in Figures 5 and 6.

## Discussion

The presence of Ca^2+^ ions perturbs the CD spectrum of **1** only slightly after 1 equivalent of CaCl_2_ is added to the peptide sample (Figure 1). No significant difference between the CD spectra collected before and after CaCl_2_ addition for synthetic peptides models of the calcium-binding site in α-lactalbumin was also observed by Kuhlman and co-workers [22]. These results were interpreted as an indication that either the studied peptides did not bind calcium or, if they did, calcium binding did not induce additional structure. Our NMR results for the effect of calcium on **1** provide evidence of Ca^2+^ chelation through changes in the chemical shifts of key amino acids (*vide infra*). Therefore, we interpret the slight change in the CD spectra detected herein as an indication that the structure of **1** is not altered significantly by Ca^2+^ binding. This assumption is also supported by the NOESY spectra collected for **1** and **1** + Ca^2+^ (Figure 4), which show the same non-trivial NOE signals for both versions of **1**. In the Kuhlman *et al.*[22] investigation, the addition of trifluoroethanol to the peptide samples induced them to adopt a helical conformation, which led to stronger Ca^2+^ binding and, therefore, more noticeable differences between the CD spectra of these peptides before and after CaCl_2_ addition. A left-handed helical conformation for the *P. falciparum* CSP has been previously proposed [13]. Based on this possibility, it could be expected that Ca^2+^ binding by a peptide fragment of the *P. falciparum* CSP with helical tendencies stronger than those of **1** would show very different CD spectra before and after CaCl_2_ addition.

The slight change in the CD spectrum of **1** upon Ca^2+^ addition could also be interpreted as an ionic-strength effect due to non-specific ionic interactions between the acidic D residues and the Ca^2+^ ions. However, Verdini *et al.*[15] found no effect on the CD spectra of *P. falciparum* CSP peptides when collected under high concentrations of sodium and potassium chloride salts.

The CD spectrum of **1** + 0.5 equivalents of CaCl_2_ is very similar to that collected for the apo-peptide. If **1** is chelating Ca^2+^ ions after the addition of 1 equivalent of CaCl_2_ to the peptide sample, it would be expected that the spectrum referred to above should appear in an intermediate position between that of the apo-peptide and the one collected for **1** + 1 equivalent of CaCl_2_. A possible explanation of the CD behavior of the Ca:**1** 0.5:1 sample is to think of the Ca^2+^-bound peptide as labile. In this case, it is expected that the metal-bound and apo-peptide exhibit chemical exchange as shown in the equation below:

(1) $${\mathrm{Ca}}^{2+}+\;\mathrm{peptide}↔{\mathrm{Ca}}^{2+}-\;\mathrm{peptide}$$

At a Ca:peptide ratio of 0.5:1 there is still significant contribution of the apo-peptide to the CD spectrum of this sample, owing to the presence of chemical exchange between the metal-free peptide and its complexed form. The addition of higher amounts of CaCl_2_ can shift the equilibrium to the right in equation (1), producing the results observed in Figure 1 for Ca:**1** ratios of 1:1, 2:1 and 4:1. This result is not surprising since the Ca affinity of many Ca-binding proteins is fine-tuned through the concentration of the ion in the cellular medium, such that biological responses initiate only at the appropriately high (or low) levels of Ca^2+^[21].

In summary, the CD results shown herein can be interpreted as an indication of weak Ca binding by **1** possibly due to lack of the required helical structure, which is triggered only at Ca:**1** ratios ≥ 1:1.

A stronger evidence of calcium binding by **1** is offered by the differences in chemical shifts (Δδ) for **1** and **1** + Ca^2+^. The magnitude of the determined Δδs is in agreement with those observed for other calcium-binding peptides [22,23]. Figure 2 indicates that calcium binding by **1** affects the signals for the protons in the C-terminal side of **1**, especially those of the D residues, which are found as participants in Ca^2+^ coordination in many Ca-binding proteins [21]. Additionally, residues V22, V14, and N25 seem to also be affected by Ca^2+^ binding to **1**. The lack of significant NMR-signal shifts involving the D7-N13 segment of **1** indicates that the included amino acids do not participate in Ca^2+^ coordination.

A shown in Figure 2, residues N1, A2, N5, and V6 also exhibit shifts of their NMR signals upon addition of Ca^2+^ to **1**. We attribute these shifts to a change of the magnetic and/or chemical environment of the involved residues upon Ca coordination by the C-terminal region of **1**, rather than to Ca binding. The reason being that, although N residues can bind Ca ions through the OH oxygen in its side chains [21], it seems unlikely that N1 and N5 would bind Ca without D7 being also part of the coordination sphere of the metal.

Portions of the overlap of the NOESY spectra collected for **1** and **1** + Ca are shown in Figure 4. It is clear from the examination of this figure that no additional non-trivial NOEs are generated in the spectrum of **1** upon Ca^2+^ addition. This result can be interpreted as an indication that **1** does not experience drastic structural changes upon calcium binding, which is also supported by our CD results (*vide supra*).

Regarding the solution conformations most likely to be found in solution for the peptide **1** + Ca, examination of Figures 5 and 6 indicates that S3 and S7 contain several of the structural elements found in the crystal structures of Ca-binding proteins. Both conformations display a Ω loop (a compactly packed segment of polypeptide chain between 6 and 16 residues in length with termini located close together, containing no regular α helices or β strands). The high frequency of Ω loops found in Ca-binding sites is a consequence of Ca binding to continuous or semicontinuous amino acid loops [21]. The MD calculations additionally depict S3 containing an Asx turn, which is a secondary structural interaction involving hydrogen bonding between the side chain oxygen atom of a D, N, S, or T residue at position *n* and the main chain NH of a residue at position *n + 2*. Various reverse turns (γ turns and β turns) are also predicted by the MD calculations within the Ω loops (Figures 5B and 6B). The presence of these loops has been considered critical in maintaining the confirmation of the ligand loop. These hydrogen-bond interactions serve to stabilize the cluster of negatively charged ligand oxygen atoms in the Ca-coordination sphere [21]. The formation of hydrogen bonds should shift the NMR signals of the residues involved. However it is possible that the intense overlap of the NMR signals observed in the spectra of **1** and **1** + Ca, due to the presence of repeated amino acids and amino acids blocks could have prevented us from detecting these shifts. In summary, the binding of calcium to **1** either in six- or seven-coordinate fashion can produce peptide conformation that are consistent with many structural features displayed by Ca-binding proteins. This fact supports the potential for calcium binding of the peptide under study.

## Conclusions

The investigation described herein strongly supports the binding of Ca^2+^ ions to **1** through the shifts of the NMR signals of key amino acids in the peptide sequence. This peptide has the same amino acid sequence as the repeat region of CSP adjacent to region I. It is therefore possible that the CSP of *P. falciparum* can bind Ca^2+^ ions even more efficiently than **1**, since this protein is proposed to have a helical structure [13]. Examination of the structural calculation results indicates that peptide **1** + Ca has the potential to assume solution conformations that are compatible with the NMR results, and consistent with several structural features exhibited by many Ca-binding proteins.

The role of Ca^2+^ ions in the frame of CSPs needs to be understood. Peptide **1** in *P. falciparum* CSP is very close to region I. This region is important for parasite infectivity [24]. It is possible that the structural tendencies prompted by Ca^2+^ coordination could generate cooperative effects that can propagate to region I, and generate the appropriate conformation for interaction with hepatocyte receptors. On the other hand, dynamic light scattering, velocity sedimentation and high resolution atomic force microscopy studies indicated that the CSP of *P. falciparum* has a rod-like structure with a ribbon-like appearance [9]. Based on these results, the authors modelled the NANP repeat of this CSP, and found it to also be a rod-like structure about 21–25 nm in length and 1.5 nm in width. Since *P. falciparum* CSP can coordinate Ca^2+^ ions, these ions could serve to stabilize the proposed rod-like structure.

There is extensive work on the participation of Ca ions in blood-stage malaria. For example, Tanabe *et al.* demonstrated that for the murine malaria *Plasmodium chabaudi*, the concentration of Ca^2+^ progressively increases as the parasite develops inside the cell, with schizont-infected cells containing 10–20 times more calcium than the uninfected cell [25]. They also showed that there is an increased influx and a decreased efflux of calcium from the schizont-infected cell. More work developed by these authors along the same lines also demonstrated that the Ca^2+^-ATPase in the membrane of such erythrocytes operates only at 70% of that in uninfected cells [26]. It was concluded that the intra-erythrocytic parasite accumulates calcium from the cytosol of the infected cell. Additionally, cells infected by *Plasmodium berghei*[27,28] and *P. falciparum*[29] showed similar changes in Ca homeostasis. Later work also demonstrated the importance of extracellular Ca for the intra-erythrocytic life-cycle of *P. falciparum*[30]. It was shown that during the early development of the parasite, absence of calcium results in irreversible morphologically abnormal late trophozoites and parasite death. Furthermore, during the invasion process, absence of calcium resulted in reversible loss of invasive capacity [30]. It has also been proposed that invasion of the human erythrocyte by *P. falciparum* is both calcium-dependent and calcium-specific [16], and that an overload of Ca-chelators in schizont-infected erythrocytes did not inhibit parasite invasion. These results were interpreted as an indication that the role of calcium is an extracellular one. Although the research described above focuses on blood-stage malaria, the results of the work described herein indicate that Ca^2+^ ions could also affect the pre-hepatic stages of the infection. Close examination of the amino acid sequences of the repeat regions of the CSPs of various *Plasmodium* indicates that, besides *P. falciparum*, these CSPs also display plenty of possible Ca^2+^-binding sites. The acidic amino acids preferred for Ca binding are not located in the primary repeating units of most of these proteins, but rather in the alternative repeating units, which could explain their presence in the amino acid sequences of CSPs. Ca^2+^-chelation studies by CSPs other than that of *P. falciparum* are being actively pursued in our laboratory with the goal of determining their metal-chelation capabilities. The possibility that all CSPs can bind Ca^2+^ ions could have a big impact on the design and efficiency of peptide-based vaccines.

## Abbreviations

CSP: Circumsporozoite protein; CD: Circular dichroism; MD: Molecular dynamics; NMR: Nuclear magnetic resonance; NOE: Nuclear overhauser effect cross signal; NOESY: Nuclear overhauser effect spectroscopy; ps: Picosecond; rms: Root mean square; TOCSY: Totally correlated spectroscopy; δ: Chemical shift; Δδ: Difference in chemical shift.

## Competing interests

The authors declare that they have no competing interests.

## Authors’ contributions

ET prepared the NMR samples, collected and analysed the NMR data, performed the molecular dynamics calculations, and contributed to the draft paper. TL was principal investigator. Both authors read and approved the final manuscript.
